# Decision-making model for production and operation of underground gold mines considering low-carbon condition

**DOI:** 10.1038/s41598-025-88762-2

**Published:** 2025-02-04

**Authors:** Jie Hou, Yingyu Gu, Guoqing Li, Guangjun Guo, Qianqian Yu

**Affiliations:** 1 School of Civil and Resource Engineering, University of Science and Technology Beijing, Beijing, China; 2 Baosteel Resources Holding (Shanghai) Co., Ltd., Shanghai, China; 3 Jiaojia Gold Mine, Shandong Gold Group Mining (Laizhou) Co., Ltd., Yantai, China

**Keywords:** Underground gold mines, Low-carbon transition, Carbon tax, Operation decisions, System dynamics simulation, Environmental impact, Engineering

## Abstract

Within the framework of a low-carbon transition and integrated mineral resource exploitation, this study presents an innovative system dynamics (SD) model designed to optimize decision-making and enhance profitability in underground gold mining operations. The novel approach seamlessly integrates critical subsystems, including reserves, mining, ore dressing, smelting, financial, and carbon reduction, offering a comprehensive framework for the analysis of efficiency and sustainability. Utilizing causal loop and system flow diagrams, the model elucidates the synergistic impacts of index variations on mine operational efficiency. The model is applied to a case study involving three mining areas within a specific gold mine in China, where sensitivity analysis identifies key indicators affecting profitability. Furthermore, it examines dynamic trends under varying carbon tax scenarios. The findings reveal that mining strategic adjustments can significantly enhance profitability, extend the operational lifespan of mines, and reduce emissions.

## Introduction

The global push for sustainable development and carbon emission reduction has reshaped the mining industry^1–3^. Initiatives such as the “Green Mining Initiative,” launched by the International Energy Agency (IEA) and the Organization for Economic Co-operation and Development (OECD), and national commitments to carbon neutrality, including China’s “carbon peaking” and “carbon neutrality” goals, emphasize the urgent need for low-carbon transitions in mining operations. These policies have promoted governments and enterprises worldwide to adopt low-carbon strategies, fundamentally altering the operational framework of the mining sector^4–7^.

Traditional mining operations, once primarily focused on maximizing economic output, are now evolving to integrate environmental stewardship into core practices^8,9^. In the context of low-carbon development, modern mining systems prioritize resource efficiency, energy optimization, and emissions reduction, particularly in areas such as resource exploitation and tailings management^10–13^. This shift has introduced significant complexity into operational decision-making, requiring enterprises to balance profitability with sustainability goals^14–16^.

In the transition to a low-carbon economy, scholars such as Safa et al. and Gorman and Dzombak have highlighted the critical role of integrating clean energy and advancing mining technologies to significantly reduce carbon emissions and energy consumption, thereby mitigating environmental impacts^17,18^. These efforts are complemented by stringent resource management and the adoption of low-carbon principles, crucial for achieving resource efficiency and minimizing waste in sustainable mining practices.

Addressing the challenges of the low-carbon economy in mining requires an understanding of complex interconnections within underground metal mines. System Dynamics (SD), introduced by Forrester, is a robust simulation-based method adept at handling complex feedback systems, widely used in production planning and operations management^19–21^. Its applicability to project-oriented industry chains has been emphasized by Shamsuddoha and Woodside, while Jamalnia and Feili have utilized it in manufacturing operations analysis^22,23^. Patroklos and Charalampos demonstrated its positive impacts on sales performance with an SD prototype for real-time manufacturing, and Georgiadis developed an SD model for capacity planning in the resource recovery industry, focusing on enhancing corporate profits and recycling company productivity^24,25^.

In mining, SD has been employed to model environmental and economic interactions, forecast long-term metal production, and predict enterprise profits^26–28^. Mondoukpè Lagnika et al. developed an integrated tool for managing mining environmental impacts, with Itumeleng et al. simulating gold mining’s environmental effects^29^. Studies by Itumeleng and Amir et al. have evaluated environmental costs in open-pit mining^30,31^. Aranoglu et al. explored the supply chain dynamics of informal and illegal gold mining in the Amazon, while Lim et al. introduced a dynamic simulation approach for deep-seabed mining, assessing comprehensive environmental impacts to tackle extreme resource exploitation challenges^32,33^.

Despite substantial progress in low-carbon mining research, several critical gaps persist in the literature. First, many studies tend to focus on isolated components of the mining sector, such as single-variable analyses or specific technological innovations, without addressing the complex interdependencies between environmental, economic, and operational factors. While considerable efforts have been made to model the environmental impacts of mining, these studies often overlook the dynamic feedback mechanisms and the long-term effects of policy interventions, such as carbon taxes or emission regulations. Additionally, most existing models predominantly concentrate on surface mining or specific mineral types, neglecting the unique challenges posed by underground metal mining operations. These challenges include the intricate processes of energy optimization, resource efficiency, and emissions management within this context. Collectively, these limitations highlight the necessity for a more integrated, dynamic modeling approach to mining operations within low-carbon scenarios.

Research has demonstrated that leveraging clean energy, improving production processes, and adopting advanced technologies can significantly reduce the carbon footprint of mining operations. Additionally, low-carbon mining frameworks emphasize resource-efficient utilization and pollution mitigation, creating opportunities for the industry to enhance both economic and environmental outcomes. However, the intricate interconnections and feedback loops within mining systems, particularly in underground metal mines, remain underexplored and require further investigation.

To address these gaps, this study introduces an innovative application of the System Dynamics (SD) methodology to model and analyze the production and operational processes of underground metal mines in low-carbon scenarios. Unlike prior studies that focus on specific regions, mining types, or short-term outcomes, this research adopts a comprehensive, system-level approach that captures the complex interdependencies between environmental, economic, and operational factors. By constructing a robust technical and economic indicator system, which integrates critical carbon reduction variables such as emissions levels, carbon tax rates, and their associated costs, this study provides a dynamic simulation framework for evaluating and optimizing mine operations under a range of low-carbon policy scenarios. This integrated modeling approach is a significant departure from existing research and provides actionable insights for mining enterprises seeking to balance profitability with sustainability goals.

## Analysis of mine operation considering low-carbon transition

### System of technical and economic indexes

**Fig. 1:**
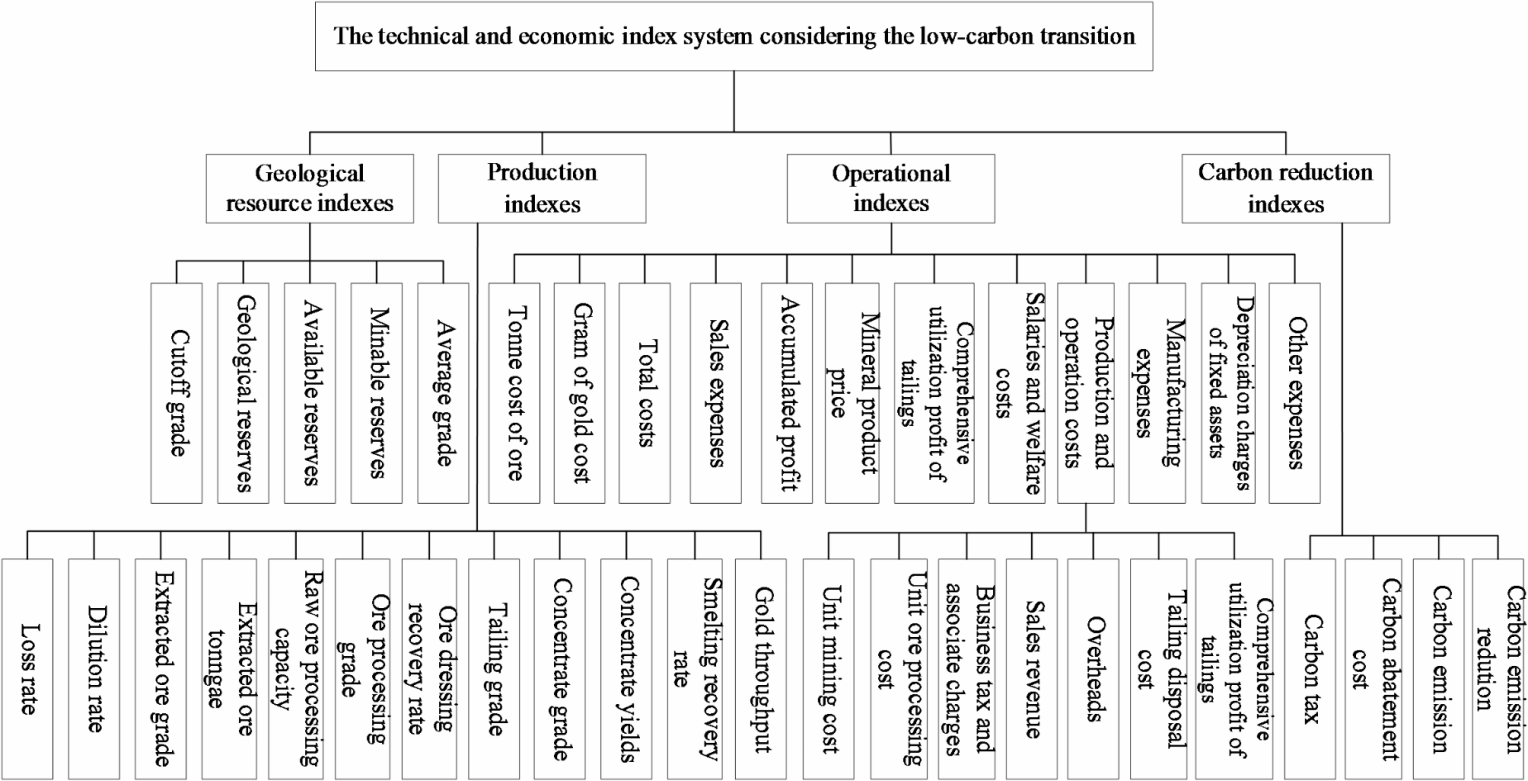
The technical and economic index system considering the low-carbon transition.

The technical and economic index system is built on the principles of demand orientation, data accessibility, and index relevance and hierarchy. These principles ensure that the indicators are meaningful, measurable, and accurately reflect the operational and environmental performance of underground gold mining within a low-carbon framework. The system is tailored to meet the needs of mining enterprises transitioning to low-carbon operations, effectively balancing profitability, resource efficiency, and carbon reduction. Indicators are derived from accessible data, including geological surveys, production records, and financial reports, allowing for precise quantification and analysis, ultimately providing decision-makers with actionable insights. The index system encompasses four key areas: geological resource indexes, production indexes, operational indexes, and carbon reduction indexes, as illustrated in Fig. 1.

### Analysis of interrelated indexes influencing system efficiency

The technical and economic indexes affecting the efficiency of mine operation are dynamically correlated and exhibit complex feedback relationships. A change in one index can trigger a cascade of changes in other indexes, potentially leading to a counter-effect on the original index^34^. The system of technical and economic indexes designed to facilitate the low-carbon transition in metal mine operations has the following characteristics:

1. The sub-index system for mineral resource utilization focuses on geological reserves, production technology, and operational indexes. It includes indexes such as ore processing and smelting recovery rates, ore processing grade, tailing grade, minable reserves, loss and dilution rates, extracted ore tonnage, and raw ore processing capacity. These indexes help assess the comprehensive utilization efficiency and integrated exploitation of mineral resources.
2. The sub-index system for low-carbon economy requirements includes carbon tax costs, carbon abatement costs, carbon emissions, and carbon emission reductions as carbon reduction indexes. The extraction and transportation of ore necessitate the use of large-scale machinery, involving the consumption of fuels, electricity, and explosives, all of which contribute to energy usage and, consequently, carbon emissions. According to the IPCC, carbon emissions can be calculated based on the amount of energy consumed and the associated carbon emission factors. In efforts to mitigate carbon emissions, mines implement strategies such as reducing solid waste disposal, utilizing clean energy sources, and conducting land reclamation.
3. The sub-index system for financial indicators considers the enterprise revenues, costs, and profits generated by production activities in the above two parts. Costs are classified in detail, and sales revenue and profit indexes are added, considering market mineral product prices to describe the economic benefits of mines and reflect the operation level of mining enterprises.

### Research framework and modeling premise

**Fig. 2:**
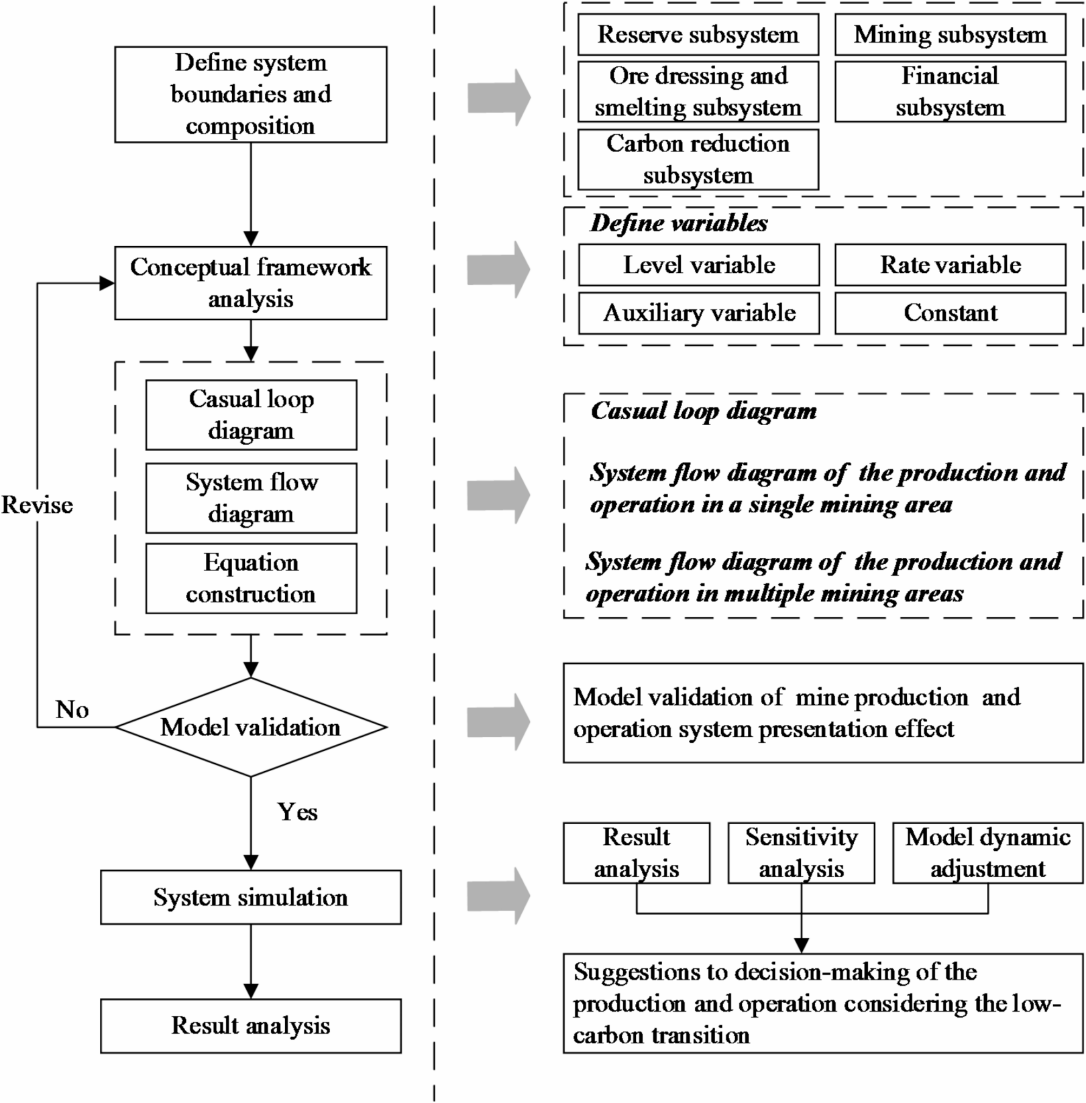
Modeling steps and research framework.

A System Dynamics (SD) model is constructed to simulate the dynamic effects of various conditions to determine the optimal solution schemes for mining enterprises^35–37^. The modeling steps and research framework are illustrated in Fig. 2.

Many indexes are affected by other factors and change over the service time of the mining area. The functional relationships between the relevant technical and economic indexes are different due to the differences in mineral resources and mining operations in different mining areas. Thus, the relevant equations need to be established based on historical data from actual metal mine production operation situation.

## Construction of system dynamics model

### Definition of system boundaries and variables

In constructing the SD model, it is crucial to define the system boundaries and variables with precision to ensure accurate simulation of underground gold mining operations under low-carbon conditions. The model incorporates several subsystems—reserves, mining, ore processing and smelting, financial, and carbon reduction—each interacting within defined boundaries to reflect the real-world complexity of mining enterprises.

1. System Boundaries: The model boundaries encompass the entire operational framework of underground gold mining, focusing on processes impacting carbon emissions and resource utilization. By defining these boundaries, the model captures both internal dynamics and external regulatory and market conditions.
2. Level Variables: Stock variables like total available reserves and accumulated profit accumulate over time, offering a snapshot of resource and financial status.
3. Rate Variables: These include variables such as the decrease in available reserves and sales revenue, essential for understanding the dynamics of resource depletion and financial flows.
4. Auxiliary Variables: Supportive variables such as geological reserves and ore processing grade assist in evaluating resource potential and processing efficiency.
5. Constants: Fixed values, like smelting recovery rate and unit mining cost, provide model stability by representing unchanging operational parameters.

The main variables are provided in Tables 1, 2, 3, 4, 5 and 6. The currency utilized is Chinese RMB (¥).

**Table 1:** Set list.

Set	Description
*i*	Set of multiple mining areas

**Table 2:** Main variables of the reserve subsystem.

Symbols	Description	Unit	Type
${Q_a}$	Total available reserves	10^4^ t	Level
${Q_{{a_i}}}$	Available reserves *i*	10^4^ t	Level
${Q_d}$	Decrease in total available reserves	10^4^ t	Rate
${Q_{{d_i}}}$	Decrease in available reserves *i*	10^4^ t	Rate
${Q_i}$	Geological reserves *i*	10^4^ t	Auxiliary
${Q_{{m_i}}}$	Minable reserves *i*	10^4^ t	Auxiliary
${g_i}$	Cut-off grade*i*	g/t	Auxiliary
${g_{{k_i}}}$	Average grade *i*	g/t	Auxiliary

**Table 3:** Main variables of the mining subsystem.

Symbols	Description	Unit	Type
${Q_{{t_i}}}$	Annual extracted ore tonnage*i*	10^4^ t/year	Auxiliary
${Q_{{s_i}}}$	Stope production capacity *i*	10^4^ t/year	Constant
${T_i}$	Service time *i*	year	Auxiliary
${\mu _i}$	Loss rate *i*	%	Auxiliary
${\rho _i}$	Dilution rate *i*	%	Auxiliary
${g_{{e_i}}}$	Extracted ore grade *i*	g/t	Auxiliary

**Table 4:** Main variables of the ore dressing and smelting subsystem.

Symbols	Description	Unit	Type
${Q_h}$	Ore processing plant production capacity	10^4^ t/year	Constant
${Q_r}$	Raw ore processing capacity	10^4^ t/year	Auxiliary
${Q_{{r_i}}}$	Raw ore processing capacity *i*	10^4^ t/year	Auxiliary
${g_h}$	Ore processing grade	g/t	Auxiliary
${\gamma _h}$	Ore dressing recovery rate	%	Auxiliary
${\gamma _c}$	Smelting recovery rate	%	Constant
${\gamma _s}$	Cyanide recovery rate	%	Constant
${M_c}$	Concentrate yields	t	Auxiliary
${M_t}$	Tailing yields	t	Auxiliary
${g_m}$	Concentrate grade	g/t	Constant
*M*	Gold throughput	kg	Auxiliary

**Table 5:** Main variables of the financial subsystem.

Symbols	Description	Unit	Type
${P_a}$	Accumulated profit	10^4^ RMB	Level
*R*	Sales revenue	10^4^ RMB/year	Rate
*C*	Total costs	10^4^ RMB/year	Rate
*K*	Fixed costs	10^4^ RMB/year	Constant
${C_p}$	Operation costs	10^4^ RMB/year	Auxiliary
${P_g}$	Gold price	RMB/g	Auxiliary
${S_t}$	Business tax and associate charges	10^4^ RMB	Auxiliary
${C_o}$	Overheads	RMB/t	Constant
${C_s}$	Sales expenses	RMB/t	Constant
${C_f}$	Tonne cost of ore	RMB/t	Auxiliary
${C_g}$	Gram of gold cost	RMB/g	Auxiliary
${C_{{m_i}}}$	Unit mining cost *i*	RMB/t	Auxiliary
${C_h}$	Unit ore processing cost	RMB/t	Auxiliary
${g_t}$	Tailing grade	g/t	Constant
${C_d}$	Tailing disposal cost	10^4^ RMB/year	Auxiliary
${C_d}^{o}$	Unit tailing disposal cost	RMB/t	Constant
${C_u}$	Comprehensive utilization cost of tailings	10^4^ RMB/year	Auxiliary
${C_u}^{o}$	Unit comprehensive utilization cost of tailings	RMB/t	Constant
${P_u}$	Comprehensive utilization profit of tailings	10^4^ RMB/year	Auxiliary
${P_u}^{o}$	Unit comprehensive utilization profit of tailings	RMB/t	Constant

**Table 6:** Main variables of the carbon reduction subsystem.

Symbols	Description	Unit	Type
*w*	Unit carbon tax price	RMB/t	Auxiliary
${J_i}$	Carbon abatement cost *i*	10^4^ RMB/year	Auxiliary
$W({Q_{{r_i}}},{J_i})$	Carbon emission volume *i*	10^4^ t	Auxiliary
$\Delta {e_i}$	Carbon emission reduction volume *i*	10^4^ t	Auxiliary
$\beta$	Additional environmental benefits per unit of carbon emission reduction	RMB/t	Constant
$\:\pi\:$($\Delta {e_i}$)	Additional environmental benefits *i*	10^4^ RMB/year	Auxiliary

### Analysis of system dynamics model

**Fig. 3:**
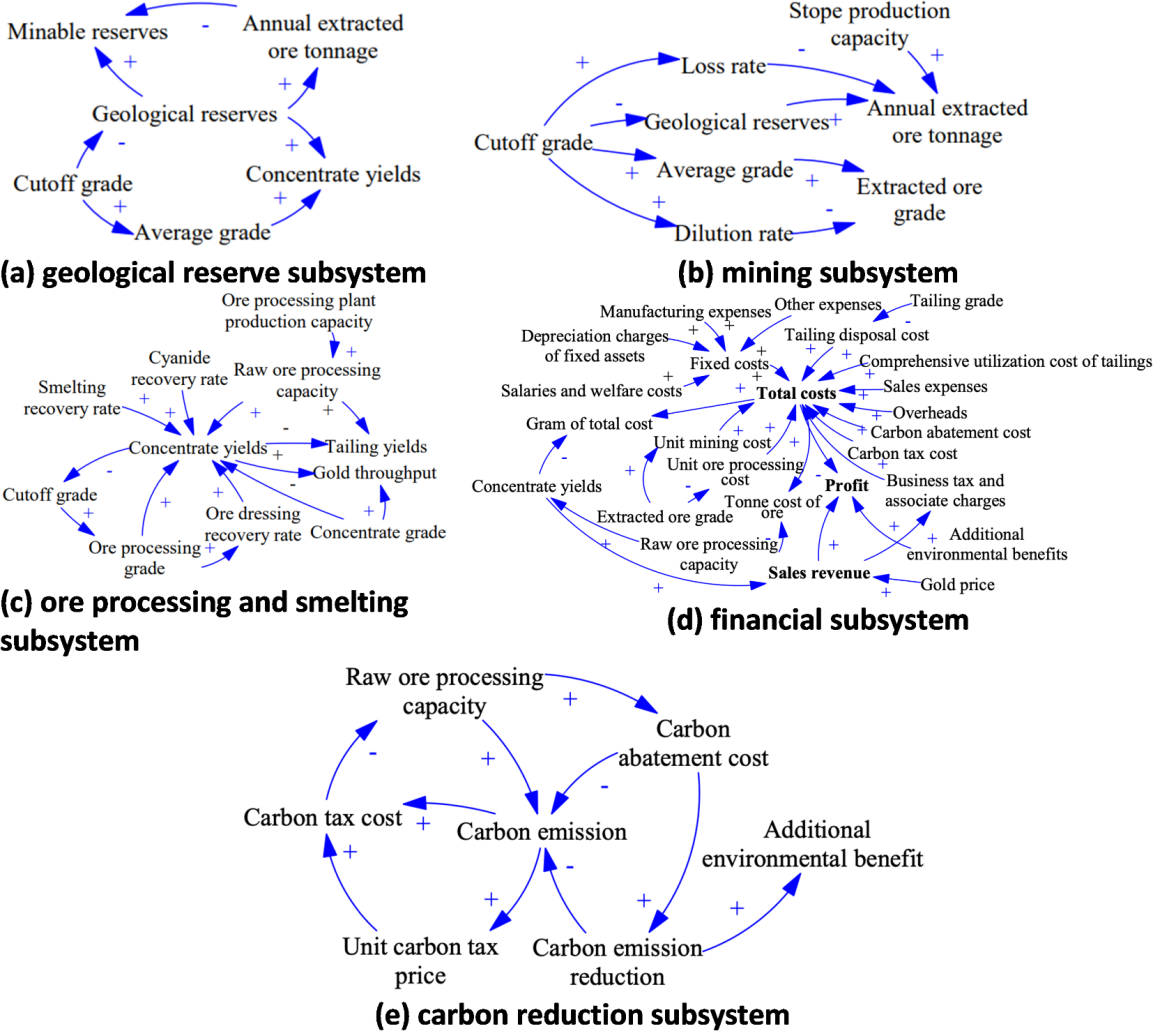
Causal loop diagrams of five subsystems. (**a**) Reserve subsystem; (**b**) mining subsystem; (**c**) ore processing and smelting subsystem; (**d**) financial subsystem; (**e**) carbon reduction subsystem.

The causal loop diagram enables a qualitative analysis of the system structure and causal feedback relationships between indexes within the system. The positive or negative sign of the arrow indicates a change in the same direction or a change in the opposite direction of two variables respectively. The Vensim PLE software is applied to describe and construct the causal loop diagram of reserves, mining operation, ore dressing and smelting, finance and carbon reduction of mines, which is illustrated in Fig. 3. As can be seen from Fig. 3, there are complex interactions between indexes of the operation system of a gold mining enterprise. One index may be affected by other indexes of this subsystem or another subsystem. For instance, changes of cut-off grade could affect several indexes such as geological reserves, loss rate and average grade. Concentrate yields could be influenced by several indexes such as ore processing grade, ore dressing and smelting recovery rate, concentrate grade and raw ore processing capacity simultaneously.

**Fig. 4:**
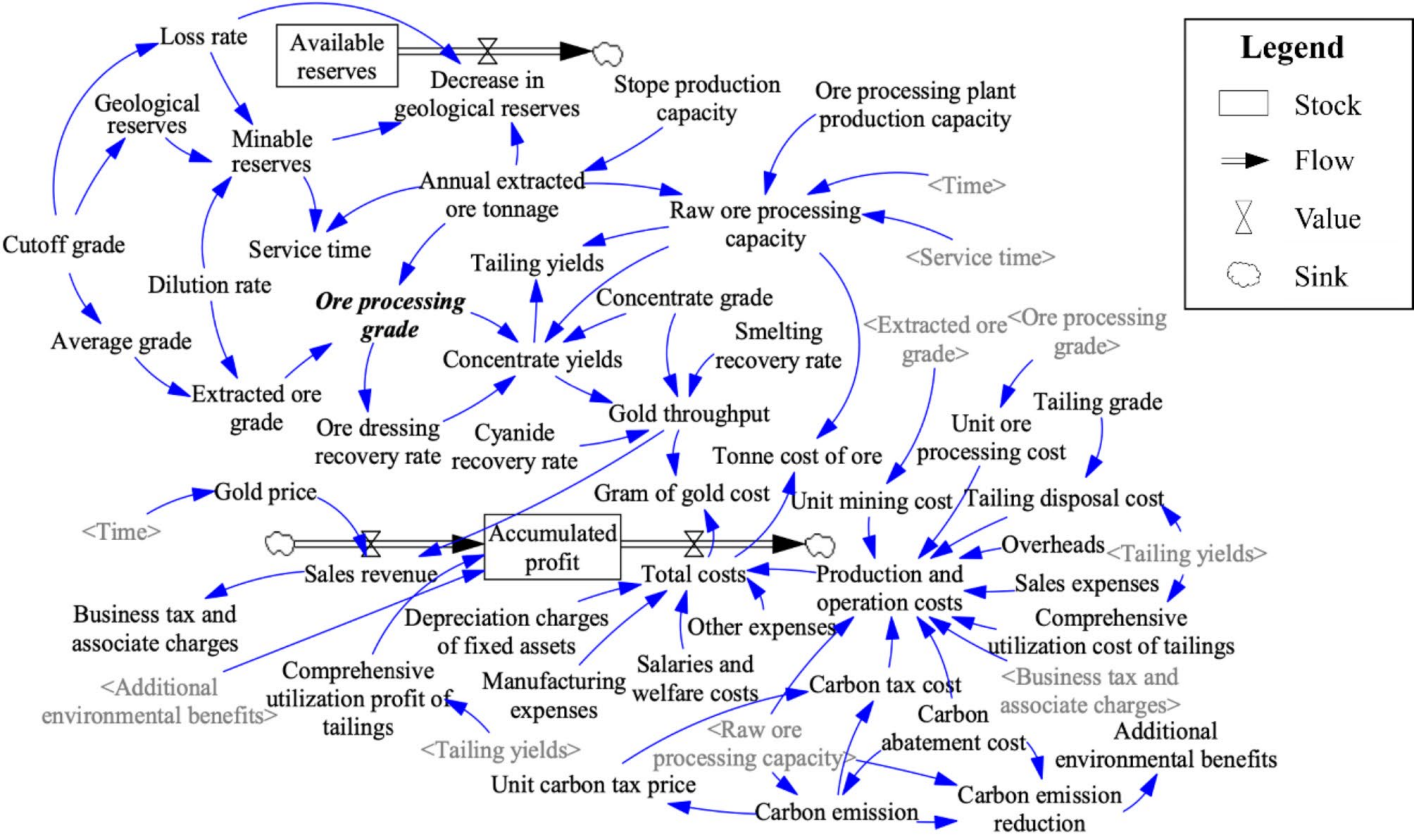
The system flow diagram of the operation in a single mining area.

In order to predict the production and operation effect of the mine under the influence of carbon tax policy in a certain period of time, a system flow diagram is built based on the causal loop diagrams above to quantify the logical relationship between key indexes. The simulation model forms a carbon reduction subsystem according to the carbon feedback loop, which is helpful to analyze the changes of indexes under the carbon emission reduction strategy. The system flow diagram of the operation effects of every sector in a single mining area is shown in Fig. 4.

**Fig. 5:**
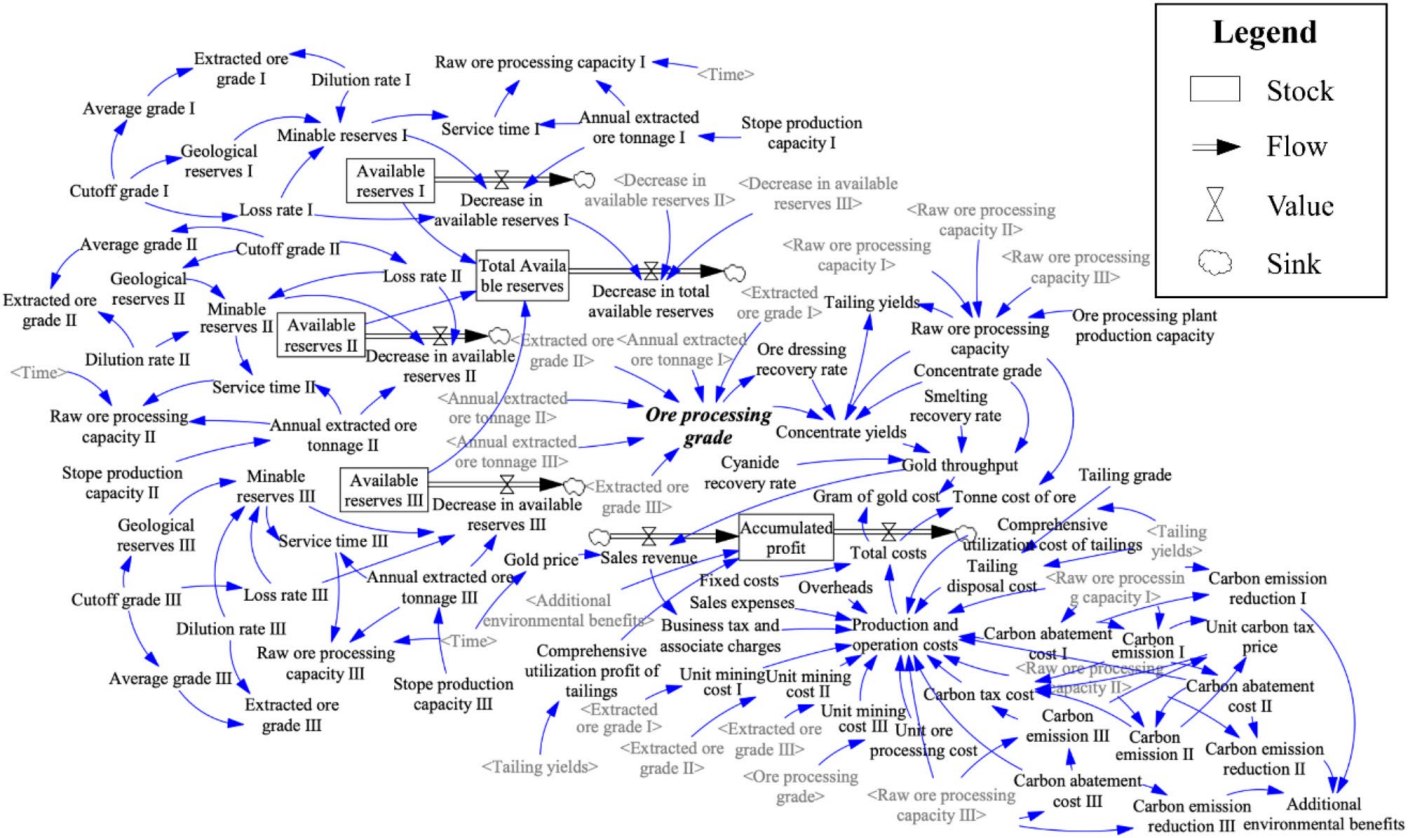
The system flow diagram of the operation in multiple mining areas.

A systematic flow diagram, as shown in Fig. 5, has been developed based on a gold mine named JiaoJia in China. The model has been refined to accommodate the three separate mining areas within the mine. This diagram is designed to facilitate operational simulation analysis across these mining areas, with consideration given to the low-carbon transition at each stage of the industry chain.

### Equation determination

**Fig. 6:**
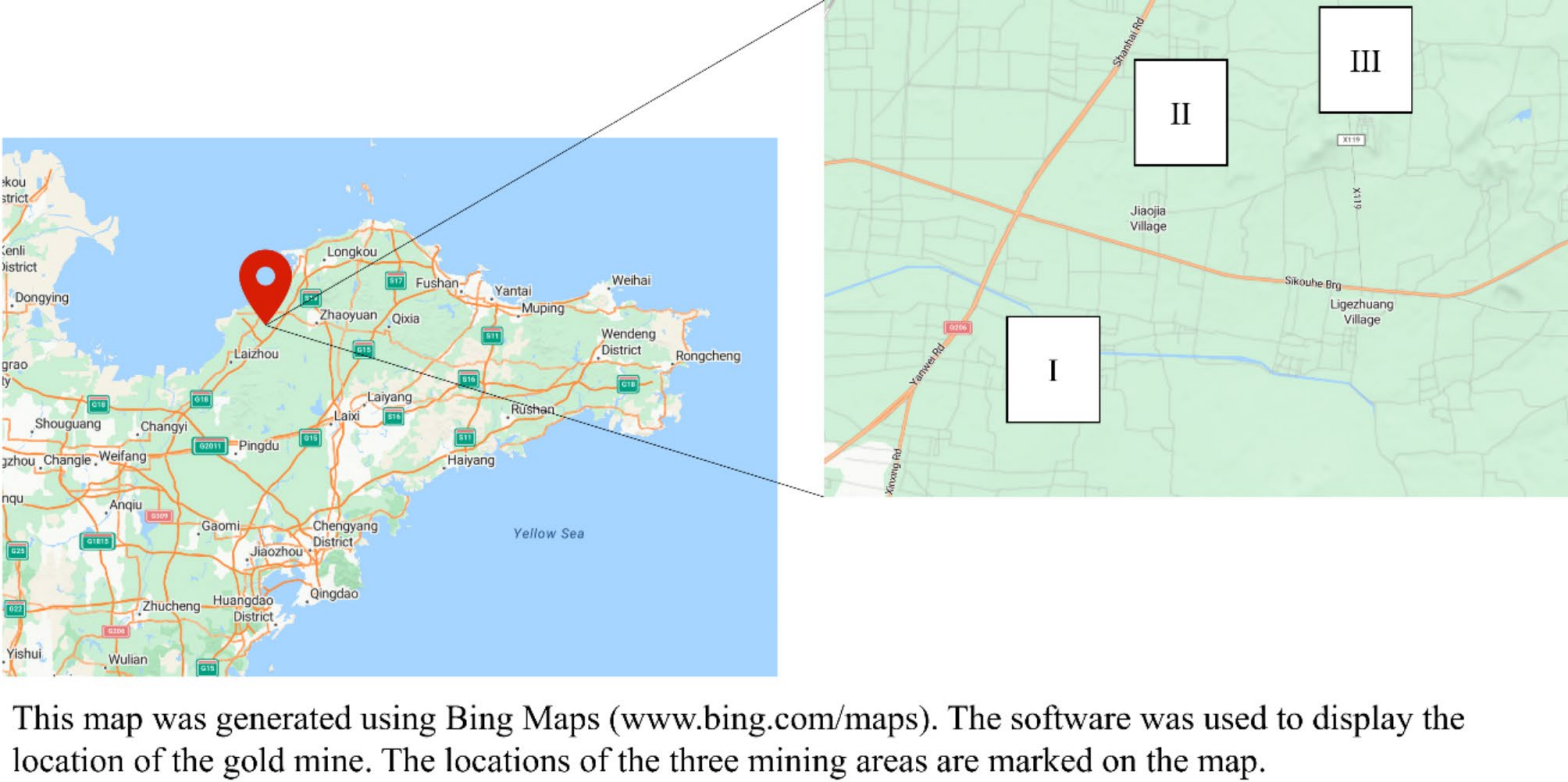
A gold mine located in China.

The research background of this study is based on three mining areas operated by a gold mining company in China shown in Fig. 6, which actively advocates the integrated exploitation of mineral resources. Mining areas I, II and III are all currently in a stable production stage. Each mining area possesses its own complete mining system, operating independently. However, these mining areas share a common ore processing plant, where extracted ores are transported for beneficiation operations. In fact, there are differences between theses mining areas in terms of resource reserves, production capacity, ore grade conditions, costs and output of minerals, etc.

**Fig. 7:**
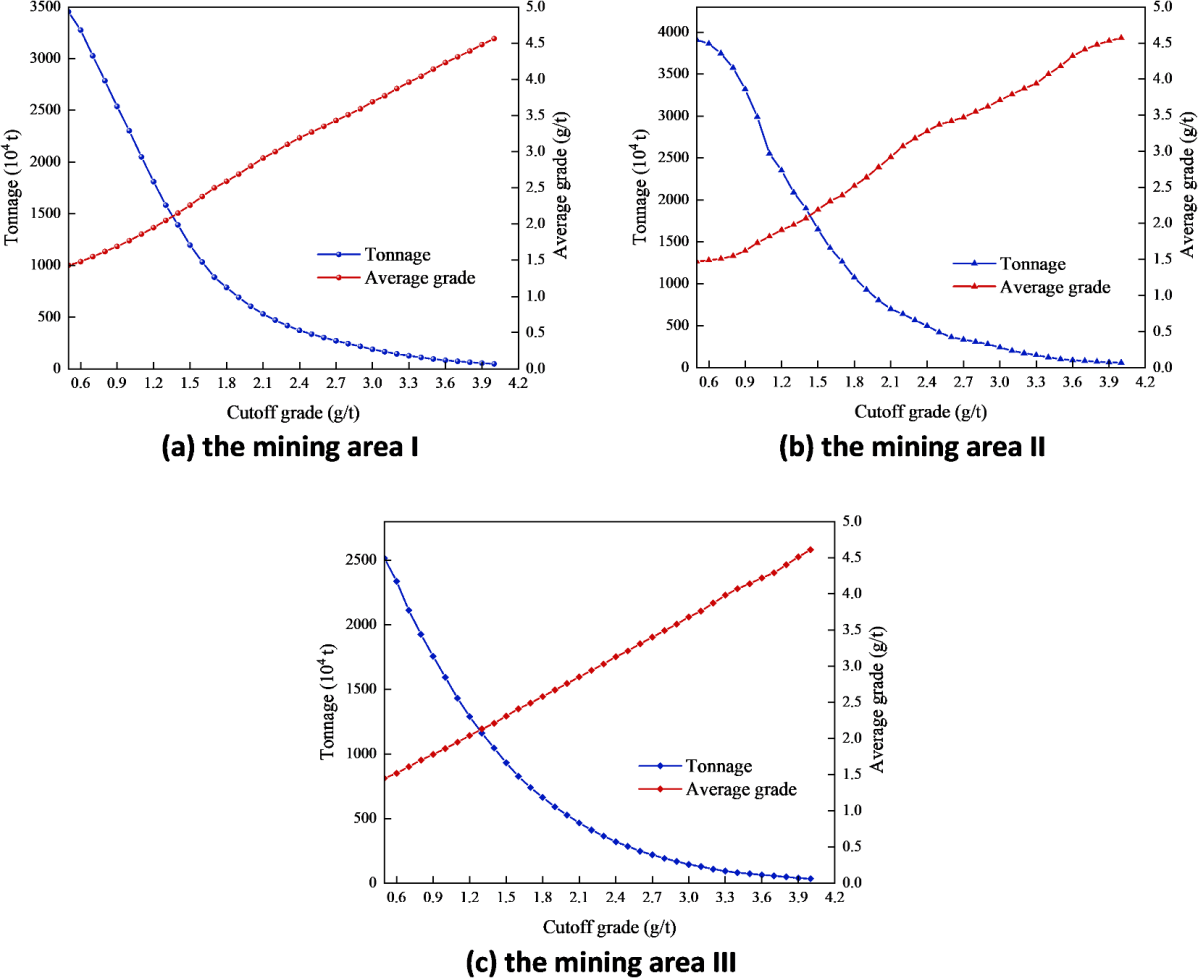
Grade and tonnage curve charts of geological resources in three mining areas. (**a**) The mining area I; (**b**) the mining area II; (**c**) the mining area III.

Corporate decision makers hope to maximize the economic and environmental benefits of enterprises in the influence of the carbon tax while meeting the market demand of overall gold throughput. Therefore, the operation decision analysis should be required for three mining areas considering the carbon emission reduction policy. The grade and tonnage curve chart of geological resources in mining areas I, II and III respectively is illustrated in Fig. 7.

Variables in this SD model are defined through equations or values. The table function of the gold price is based on the gold price statistics over past ten years. Referring to the 2023 Carbon Pricing Mechanism Development status and Future Trends report released by the World Bank, it is assumed that the model adopts a progressive unit carbon tax price scheme^38^. The carbon abatement costs refer to the additional capital and technical expenditure invested to reduce carbon emissions, which is the basis of the determination of carbon tax. In addition, other parameters are quantified based on data generated from actual operations in three mining areas under a gold mine in China.

The equations illustrating the relationships between the various indicators are presented in Table 7. Detailed explanations of the specific meanings of the parameters are provided in Tables 1, 2, 3, 4, 5 and 6. Especially, the related equations of level variables are integral equations, and the basic form is $INTEG(x,y)$, where *x* represents the amount of change per unit time, and *y* represents the initial value. The equation of the raw ore processing capacity is set as the conditional function. The basic form is $IF{\text{ }}THEN{\text{ }}ELSE\left( {{\text{ }}\left\{ {cond} \right\}{\text{ }},{\text{ }}\left\{ {ontrue} \right\}{\text{ }},{\text{ }}\left\{ {onfalse} \right\}{\text{ }}} \right)$, and the $cond$ represents the judgement condition, and the $ontrue$ is the value of the variable when the judgement condition is satisfied, while $onfalse$represents the value of the variable when the judgement condition is unsatisfied.

**Table 7:** Fundamental equations of five subsystems.

Category	Equations
Geological reserve subsystem	${Q_a}=INTEG(0 - {Q_d},{Q_{{m_I}}}+{Q_{{m_{II}}}}+{Q_{{m_{III}}}})$
	$${Q_{{a_i}}}=INTEG(0 - {Q_{{d_i}}},{Q_{{m_i}}}){\text{ }}i{\text{=}}I,II,III$$
	$${Q_{{d_i}}}={Q_{{t_i}}} \times {(1 - {\mu _i})^{ - 1}}$$
	$${Q_{{m_{_{i}}}}}={Q_i} \times (1 - {\mu _i}) \times {(1 - {\rho _i})^{ - 1}}$$
Mining subsystem	${g_{{e_i}}}={g_{{k_i}}} \times (1-{\rho _i})$
	$${T_i}=INTEGER{({Q_{{m_i}}} \times {Q_{{t_i}}})^{ - 1}}$$
Ore processing and smelting subsystem	${Q_{{r_i}}}=IF{\text{ }}THEN{\text{ }}ELSE(Time \leqslant {T_i},{Q_{{t_i}}},0){\text{ }}$
	$${g_h}=({Q_{{t_{_{I}}}}} \times {g_{{e_I}}}+{Q_{{t_{II}}}} \times {g_{{{\text{e}}_{II}}}}+{Q_{{t_{III}}}} \times {g_{{e_{III}}}}) \times {({Q_{{t_{_{I}}}}}+{Q_{{t_{II}}}}+{Q_{{t_{III}}}})^{ - 1}}$$
	$${M_c}={Q_r} \times {10^4} \times {g_h} \times {\gamma _h} \times {g_m}^{{ - 1}}$$
	$${M_t}={Q_r} - {M_c}$$
	$$M={M_c} \times {g_m} \times 0.001 \times {\gamma _s} \times {\gamma _c}$$
Financial subsystem	$P=INTEG(R+{P_u}+\sum\nolimits_{{i=1}}^{I} \pi (\Delta {e_i}) - C,0)$
	$$R={P_g} \times M \times 0.1$$
	$$C=K+{C_p}$$
	$${P_u}={P_u}^{o} \times {M_t} \times {10^4}$$
	$${C_f}=C \times {Q_r}^{{ - 1}}$$
	$${C_g}=C \times 10 \times {M^{ - 1}}$$
	$${S_t}=R \times 0.06$$
	$$\begin{gathered} {C_p}={C_u}+{C_d}+({C_o}+{C_s}+{C_h}) \times {Q_r}+{C_{{m_I}}} \times {Q_{{r_I}}}+{C_{{m_{II}}}} \times {Q_{{r_{II}}}}+{C_{{m_{III}}}} \times {Q_{{r_{III}}}} \hfill \\ +w \times (W({Q_{{r_I}}},{J_I})+W({Q_{{r_{II}}}},{J_{II}})+W({Q_{{r_{III}}}},{J_{III}}))+{J_I}+{J_{II}}+{J_{III}}+{S_t} \hfill \\ \end{gathered} $$
	$${C_d}={C_d}^{o} \times {M_t} \times {10^{ - 4}}$$
	$${C_u}={C_u}^{o} \times {M_t} \times {10^{ - 4}}$$
Carbon reduction subsystem	$W({Q_{{r_i}}},{J_i})=3{Q_{{r_i}}}^{2}{(8067+{J_i})^{ - 1}}$
	$$\sum\nolimits_{{i=1}}^{I} \pi (\Delta {e_i})=\beta \times \sum\nolimits_{{i=1}}^{I} {\Delta {e_i}} $$
	$$w=\left\{ \begin{gathered} {L_w},\sum\nolimits_{{i=1}}^{I} {W({Q_{{r_i}}},{J_i})} \in (0,{\chi _1}) \hfill \\ \frac{{({L_w} - {U_w})}}{{{{({\chi _1} - {\chi _2})}^2}}}{(\sum\nolimits_{{i=1}}^{I} {W({Q_{{r_i}}},{J_i})} - {\chi _2})^2}+{U_w},\sum\nolimits_{{i=1}}^{I} {W({Q_{{r_i}}},{J_i})} \in \left[ {{\chi _1},{\chi _2}} \right] \hfill \\ {U_w},\sum\nolimits_{{i=1}}^{I} {W({Q_{{r_i}}},{J_i})} \in ({\chi _2},+\infty ) \hfill \\ \end{gathered} \right.$$

In addition, initial values of the relevant technical and economic parameters of multiple mining areas in this gold mining enterprise are shown in Table 8.

**Table 8:** Initial values of the relevant technical and economic parameters.

Parameters	Units	Value	Parameters	Units	Value
${Q_{{s_I}}}$	10^4^ t/year	180	*K*	10^4^ RMB/year	87,000
${Q_{{s_{II}}}}$	10^4^ t/year	230	${C_o}$	RMB/t	27.32
${Q_{{s_{III}}}}$	10^4^ t/year	145	${C_s}$	RMB/t	3.98
${\rho _I}$	%	5.75	$\beta$	RMB/t	980
${\rho _{II}}$	%	5.73	${g_m}$	g/t	58.5
${\rho _{III}}$	%	6.60	${C_d}^{o}$	RMB/t	17.57
${\gamma _s}$	%	98.03	${C_u}^{o}$	RMB/t	20.52
${\gamma _c}$	%	99	${P_u}^{o}$	RMB/t	54.414

### Model validation and verification

To ensure the reliability and validity of the simulation model, a comparative analysis is conducted between the model’s outputs and historical production and operation data from the mining industry. The average market price of gold over the past six years is input into the simulation model using a table function. The simulation period is set for six years, and the simulation step size is set to one year. The annual cumulative profit of the case study mine is selected as the simulation variable for validation.

The simulation results are compared with the actual profit values obtained from the mine’s production and operations. As shown in Table 9, discrepancies between the simulated and actual values are consistently below 5%. This level of accuracy indicates that the model is validated.

**Table 9:** The model verification results of the mine operation.

No.	Cumulative profit (10^4^ ¥)		Error (%)
	Simulated value	Actual value	
1	101,133	96,936	4.33
2	189,933	195,768	-2.98
3	283,477	280,377	1.11
4	395,994	415,243	-4.64
5	598,636	624,071	-4.08

Therefore, it is concluded that the simulation model demonstrates a satisfactory degree of reliability and validity for the purposes of this study.

## Model simulation and result analysis

### Simulation results

**Fig. 8:**
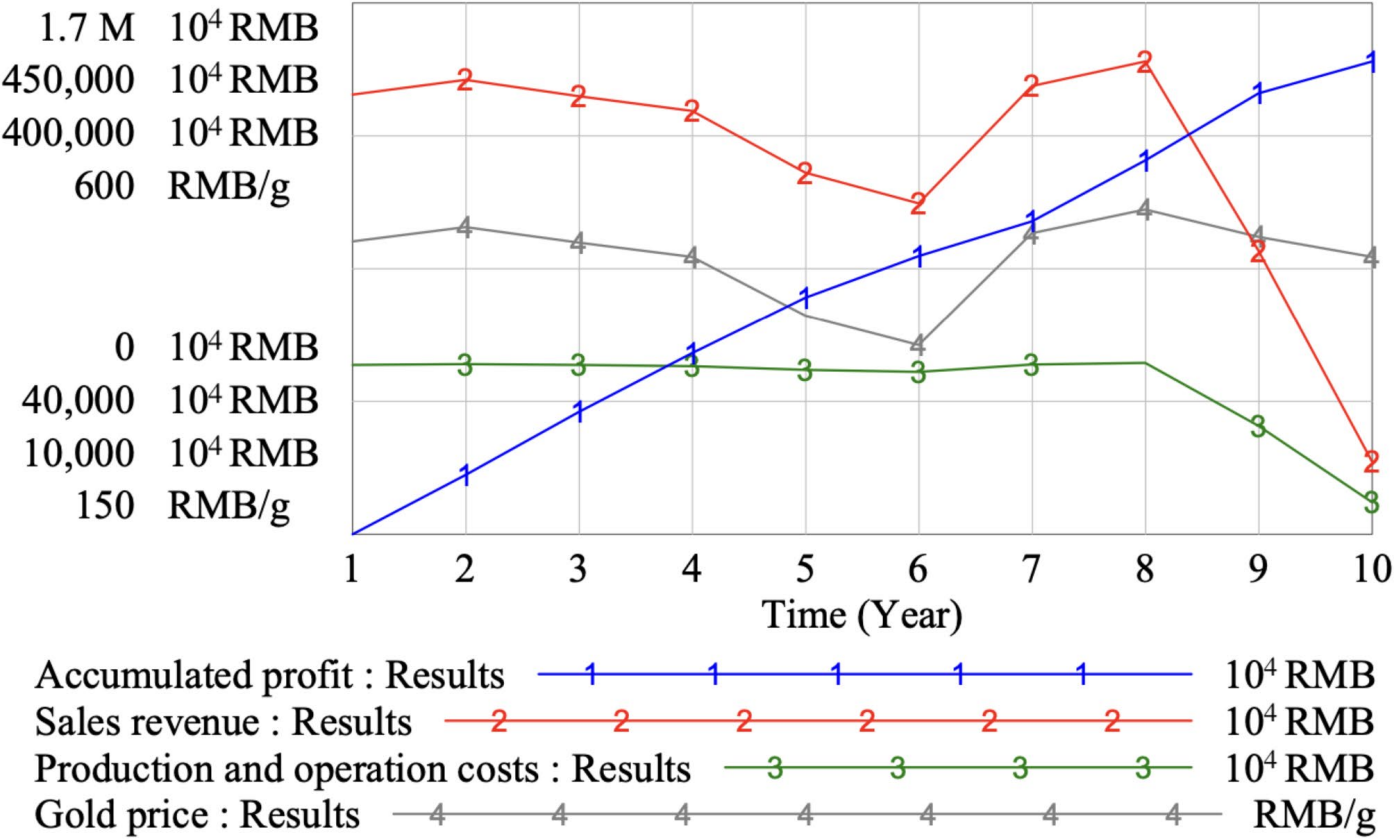
Simulation results of the annual accumulated profit, sales revenue, costs and gold price in multiple mining areas.

**Fig. 9:**
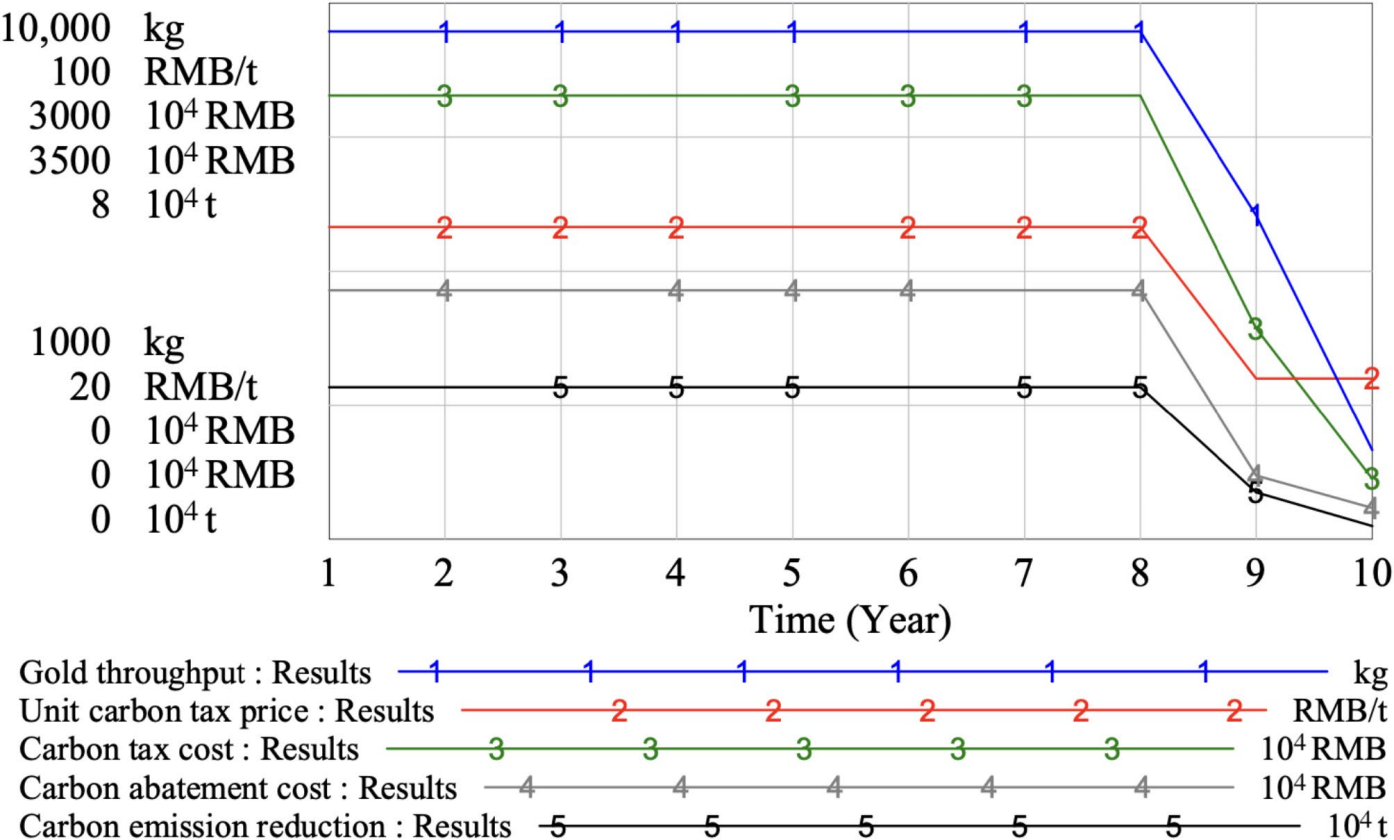
Simulation results of the gold throughput, unit carbon tax price, carbon tax cost, carbon abatement cost and carbon emission reduction in multiple mining areas.

The simulation of SD model time is set to 10 years, and the simulation time step is 1 year. The operation effect of three mining areas under a gold mine in the next 10 years is simulated. The overall production capacity of the mine is 5.55 million tons per year, and cut-off grades of three mining areas are set to 1.3 g/t, 1.2 g/t, 1.1 g/t respectively. Table 10 shows results of main technical and economic indexes obtained. Figure 8 shows simulation results of the annual accumulated profit, sales revenue, costs and gold price in multiple mining areas. The simulation results are illustrated in Fig. 9.

**Table 10:** Simulation results of main technical and economic indexes.

Variables	Units	Value	Variables	Units	Value
${g_{{e_I}}}$	g/t	1.986	${\mu _I}$	%	10.408
${g_{{e_{II}}}}$	g/t	1.868	${\mu _{II}}$	%	12.691
${g_{{e_{III}}}}$	g/t	1.827	${\mu _{III}}$	%	8.653
${T_I}$	year	8	${C_{{m_I}}}$	RMB/t	74.975
${T_{II}}$	year	9	${C_{{m_{II}}}}$	RMB/t	78.349
${T_{III}}$	year	10	${C_{{m_{III}}}}$	RMB/t	67.134
${g_h}$	g/t	1.896	${\gamma _h}$	%	93.266

According to Table 9; Fig. 7, it can be recognized that the future production service years of mine area I, II and III are 8 years, 9 years and 10 years, respectively. With fluctuations in gold prices, the annual sales revenue of the mining enterprise also has the same change. The gold price factor is one of the main factors affecting economic benefits. The accumulated profit of the mining enterprise shows a stable upward trend with time. Therefore, it is believed that under the low-carbon strategy, the operation scheme of the mine in multiple mining areas is reasonable and the efficiency is relatively stable. At the end of the 10th year, the accumulated profit of the mining enterprise can reach 1.51 × 10^10^ RMB. At the end of the 8th year, mine area I shuts down due to resource depletion, while mine area II remains operational until the 9th year. This shutdown is primarily driven by the decreasing ore reserves in mine area I. The strategic decision to close mine area I earlier than the others is based on a careful assessment of the remaining resource potential in this area. By shutting down earlier, the mining company is able to allocate resources more efficiently in the other mining areas, where ore reserves are still abundant. This also allows for a longer operational lifespan for the other areas, which contributes to more stable production in the long term. At this time, the total annual sales revenue of the mine will decrease rapidly, mainly because the annual gold throughput of the mine will decrease significantly after the shutdown^39^.

As can be seen from Fig. 8, when the three mining areas of the gold mine are all in the service life of production, the total annual gold throughput of the mine is 9523 kg. In the 9th year, since mine area I is in a state of suspension, the total annual gold throughput of the mine will decrease to 6435 kg. In the 10th year, since mine area I and II are in a state of suspension, and the total annual gold throughput of the mine will decrease to 2488 kg. The overall production scale of mine is reduced, and the corresponding carbon emission is reduced, so the carbon tax cost is also greatly reduced. In the first 8 years of the simulation period, the total annual carbon emission of the mine is about 370,000 tons, and the unit carbon tax price is 67 RMB/t at this time. From the 9th to the 10th year, the annual ore processing capacity of the mine decreases due to the discontinuation of production in the mine area, and the carbon emission of the enterprise decreases accordingly. Due to the progressive unit carbon tax price scheme, when the carbon emission is lower than 350,000 tons, the unit carbon tax price will be reduced to 44 RMB/t. In addition, due to the decrease of ore processing capacity from the 9th to the 10th year, the carbon abatement cost required by the enterprise to reduce carbon emission will be reduced, and the carbon emission reduction volume will also be reduced.

### Sensitivity analysis

**Fig. 10:**
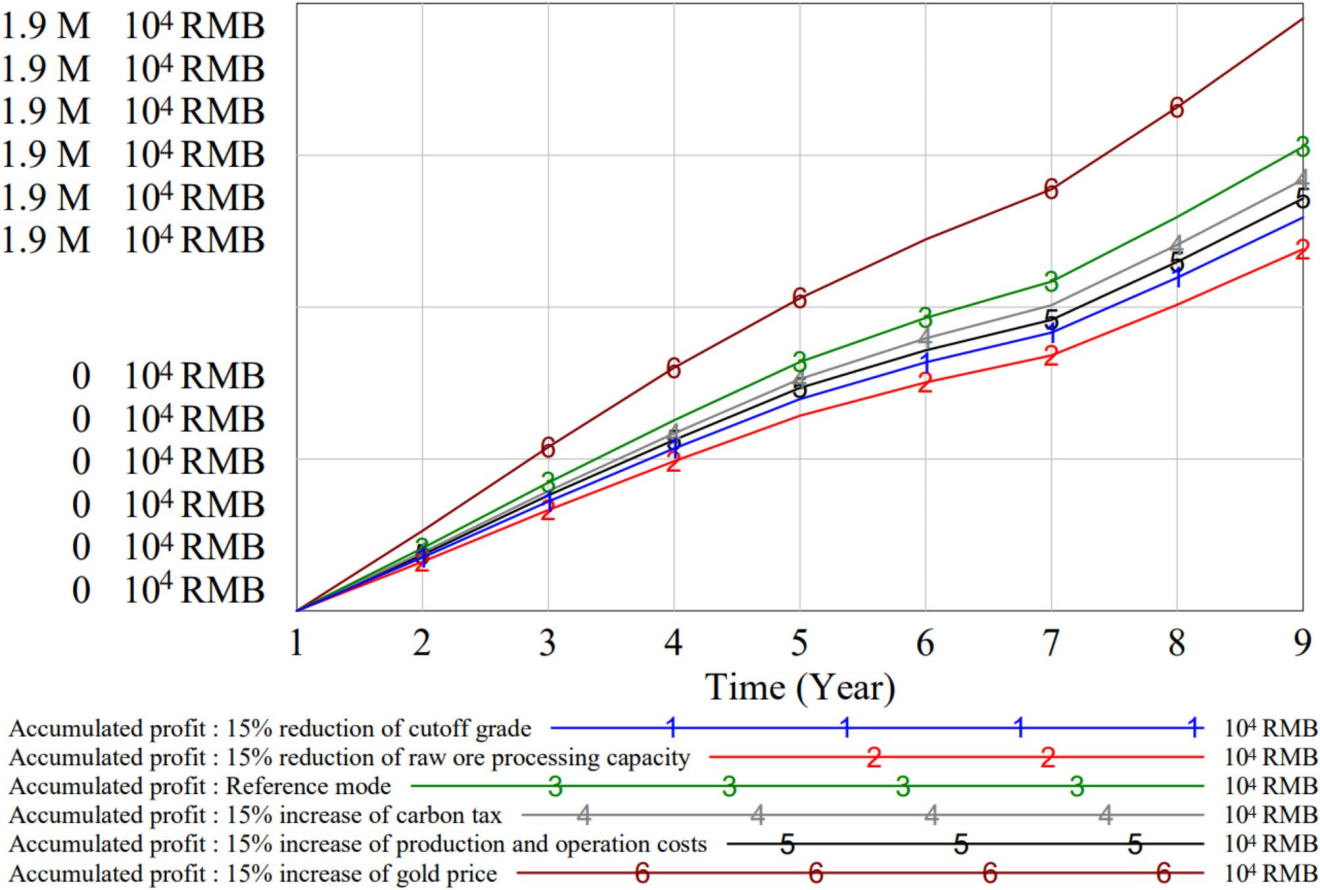
Sensitivity analysis of key indexes in this gold mining enterprise.

Sensitive parameters that influence the system’s performance are analyzed by adjusting the corresponding indexes. The results of the sensitivity analysis are illustrated in Fig. 10.

The sensitivity analysis highlights significant insights into the impact of parameter variations on the profitability and operational dynamics of mining enterprises. Among the analyzed variables, the gold price index demonstrates the highest sensitivity, indicating that even slight fluctuations in gold prices can lead to substantial changes in accumulated profits. Specifically, a 15% increase in gold prices results in nearly a 29% rise in profits over the simulation period, emphasizing the critical role of market pricing in operational decision-making.

Similarly, variations in the cut-off grade significantly influence profits. The differences in cut-off grades across the three mining areas result in varying efficiencies in resource utilization and carbon emissions. Mine area I, with the highest cut-off grade of 1.3 g/t, produces lower gold throughput but is more efficient in resource utilization, thereby consuming fewer resources per unit of gold extracted and emitting less carbon. In contrast, mine area III, with the lowest cut-off grade of 1.1 g/t, experiences higher throughput but at the expense of higher resource consumption and increased carbon emissions.

These differences in mining strategies create a synergistic effect: as lower throughput from higher cut-off grades reduces overall resource consumption and carbon emissions, focusing production on these areas can achieve a better balance between economic benefits and environmental sustainability. However, when mining areas with higher cut-off grades are phased out earlier, as seen in the case of mine area I, the remaining operational areas experience increased resource consumption and higher carbon emissions, which must be managed carefully. Therefore, an optimal strategy would involve adjusting production rates and cut-off grades in a way that minimizes both resource depletion and environmental impact while maintaining stable throughput.

The carbon tax price also exhibits a notable impact on profits, with a 15% increase leading to an 8% reduction in accumulated profits. Such findings underscore the importance of carbon tax policies in shaping enterprise strategies. Mining companies should prioritize cost-effective carbon reduction measures to mitigate the financial burden of carbon taxes while aligning with low-carbon goals.

Furthermore, operational cost increases by 15% result in an 11% decline in profits, highlighting the need for stringent cost control and operational efficiency improvements. These results suggest that enterprises should monitor and optimize operational parameters, such as ore processing capacities and energy usage, to ensure long-term economic and environmental sustainability.

**Fig. 11:**
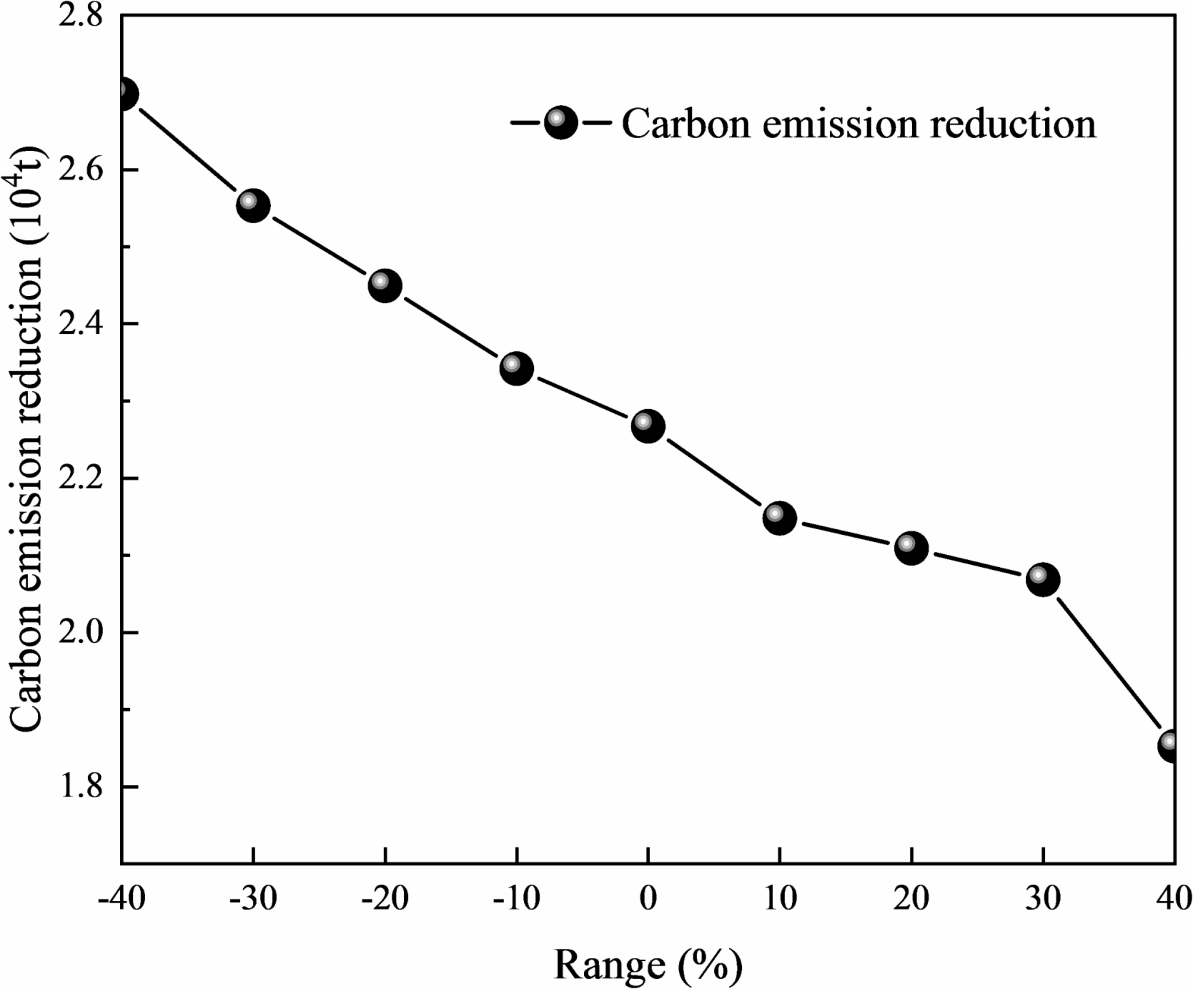
The effect of the unit carbon tax price on the mine’s optimal carbon emission reduction.

The influence of carbon tax price changes on the accumulated profit in the process of low-carbon transition in mines are considered. The price volatility of the carbon tax is set to be -40–40%. The effect of the unit carbon tax price on the mine’s optimal carbon emission reduction is illustrated in Fig. 11. Simulation results of key indexes under different unit carbon tax prices in shown in Table 11.

**Table 11:** Simulation results of key indexes under different unit carbon tax price.

Range	Carbon emission (10^4^ t/year)	Service time of the mining area (year)			Gold throughput (kg/year)
		I	II	III	
-40%	43.0	8	8	9	10,193
-30%	41.5	8	8	9	10,003
-20%	39.9	8	9	9	9830
-10%	38.7	8	9	9	9680
0	37.3	8	9	10	9523
10%	36.1	9	9	10	9364
20%	35.6	9	9	10	9297
30%	34.9	9	9	10	9218
40%	31.9	9	10	11	8771

It can be concluded that the implementation and adjustment of carbon tax policies have an important impact on the economic benefits of mines, and the carbon tax price is negatively correlated with corporate profits. When the unit carbon tax price fluctuates from − 40 to 40%, during the simulation period, the accumulated profit of the mine ranges from 1.56 × 10^10^ RMB to 1.24 × 10^10^ RMB. With the increase of the unit carbon tax price, the annual gold throughput of the mine shows a gradual decline trend, and the annual carbon emission also decreases. Due to the influence of the carbon tax policy, the mine needs to pay additional carbon abatement costs. The larger the carbon emission, the higher the unit carbon tax price, the higher the carbon tax cost of the enterprise. Therefore, in order to control the carbon emission cost and obtain better economic and environmental benefits, the comprehensive decision of the mine should consider the appropriate reduction of annual gold throughput while satisfying the stable supply of mineral products in the market. In addition, the increase of unit carbon tax price will restrict the overall production scale of the mine, and the annual ore processing capacity will decrease accordingly. In the long run, it is beneficial to extend the production service life of the mine and promote the sustainable development of mineral resources.

### Model dynamic optimization

In order to obtain the optimal operation scheme within the service life of the mine and realize the maximum economic and environmental benefits, a dynamic simulation and optimization study of the model is carried out. According to the requirements of low-carbon transition of mines, the production target of annual gold throughput of not less than 9000 kg should be met in the process of dynamic adjustment of the model. In addition, at the end of the simulation period, the accumulated profit of mines should not be less than 14.5 billion yuan, and the unit carbon tax price should not be too high.

**Fig. 12:**
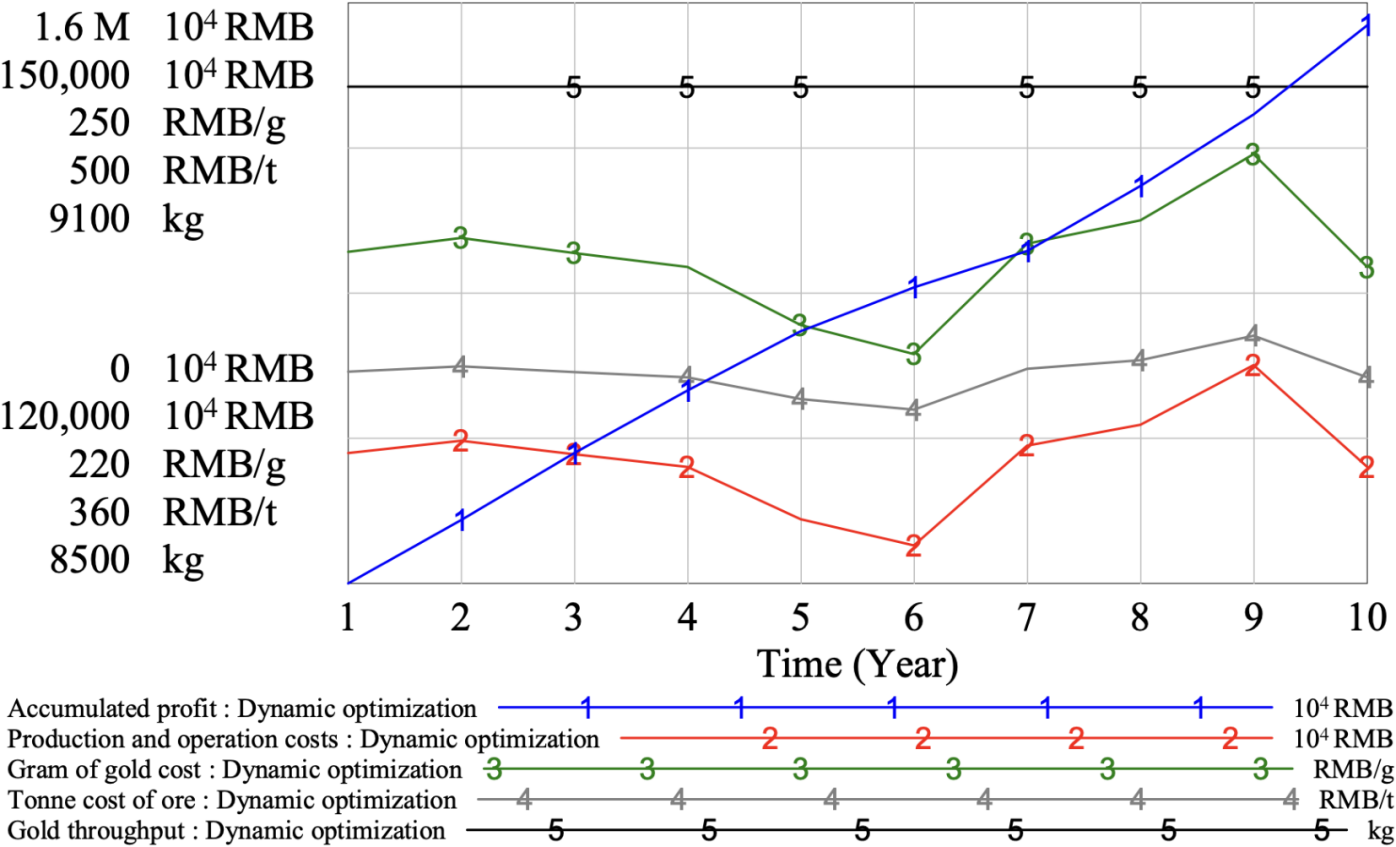
Model dynamic adjustment results.

Model dynamic adjustment results are illustrated in Fig. 12. The key indexes such as the unit carbon tax price, carbon emissions, and optimal carbon abatement costs before and after adjustment are compared, as shown in Table 12.

Combined with Fig. 12; Table 11, it can be seen that the adjusted mine cost index is relatively stable. During the simulation period, the annual gram of gold cost is stable at about 235 RMB/g, and the tone cost of ore is stable at about 400 RMB /t. At this time, the annual gold throughput of the mine is stable at 9013 kg, and the annual ore processing capacity is 5.2 million tons. The annual ore processing capacity of mine area I is 1.6 million tons, the annual ore processing capacity of mine area II is 2.15 million tons, and the annual ore processing capacity of mine area III is 1.45 million tons. Under the condition of constant resource reserves, each mine area can produce stably for 10 years at this time, which extends the production service life and realizes the stable supply of annual gold throughput. Since the carbon tax is calculated based on annual carbon emissions and increases exponentially with higher emissions, the extended service life of the optimized mining area reduces the annual operational volume, leading to lower annual carbon emissions and, consequently, reduced carbon tax payments. This production model is conducive to the sustainable utilization of mineral resources.

In addition, through dynamic adjustment, the unit carbon tax price of the mine is controlled to be stable at 44 RMB/t, the annual carbon emission is reduced from 37.3 × 10^4^ t to 32.8 × 10^4^ t, the annual optimal carbon emission reduction cost was 1476 × 10^4^ RMB, and the annual carbon emission reduction is 1.724 × 10^4^ t. At the end of the 10th year, the accumulated profit of the mine is 1.5374 × 10^10^ RMB, which is about 250 million yuan higher than that before optimization.

**Table 12:** Comparison of mine operation results.

Index	Unit	Before adjustment	After adjustment
Unit carbon tax price	RMB/t	67	44
Carbon emission	10^4^ t/year	37.3	32.8
Optimal carbon abatement costs	10^4^ RMB/year	1625	1476
Service time	year	8, 9, 10	10
Ore processing capacity	10^4^ t/year	555	520
Gold throughput	kg/year	9523	9013
Accumulated profit	10^8^ RMB	151.26	153.74

Through dynamic adjustment, the mining strategy optimizes resource extraction to align with the low-carbon goals while ensuring stable production. The optimization model ensures that the mine can maintain a steady gold throughput of 9000 kg annually, extending the mine’s service life while managing carbon emissions effectively. By dynamically adjusting the annual throughput and production rates across the mining areas, the mine can allocate resources more efficiently and lower carbon emissions, thus minimizing the financial burden of carbon taxes.

The optimization also considers the trade-offs between resource extraction and environmental impact. For instance, as the carbon tax increases, the mine adjusts its annual throughput to reduce carbon emissions and decrease the carbon tax burden. However, reducing throughput results in a slower depletion of ore reserves, which may lead to a more sustainable operation in the long run. This strategy extends the production life of the mine, ensuring that mineral resources are utilized efficiently and carbon emissions remain under control.

Moreover, by balancing the resource extraction across different mining areas, the optimized model enhances the synergy between mining areas, ensuring that areas with higher resource utilization efficiency are maximized while minimizing the environmental impact. This approach contributes to both long-term economic sustainability and low-carbon operations, promoting a sustainable mining practice.

## Discussion

This research diverges from previous studies by offering a comprehensive, system-level approach that captures these complexities. By constructing a robust technical and economic indicator system incorporating critical carbon reduction variables, we provide a dynamic simulation framework that facilitates the evaluation and optimization of mining operations under various low-carbon policy scenarios. This approach not only addresses existing gaps in the literature but also delivers actionable insights for mining enterprises aiming to align profitability with sustainability goals.

In practice, the model functions as a decision-support system, assisting enterprises in making informed choices about resource allocation, production scaling, and carbon management. By simulating various operational scenarios and carbon tax regimes, mining companies can anticipate market and regulatory changes, thereby ensuring agility and resilience in their operations. The model’s sensitivity analysis highlights the significance of key factors such as gold prices and carbon tax rates, pinpointing areas where strategic intervention can yield substantial benefits.

Nevertheless, it is crucial to acknowledge the inherent limitations of the System Dynamics (SD) model employed in this research. While SD models are adept at capturing complex system behaviors and feedback loops, they have certain theoretical and methodological constraints. A primary limitation lies in handling nonlinear relationships. In real-world mining operations, many interactions between variables are nonlinear, such as the fluctuating effects of gold prices on production costs or the relationship between carbon tax rates and emission reductions. Although the model attempts to approximate these relationships, it may not fully capture the intricate dynamics arising from highly nonlinear systems.

Additionally, the model’s flexibility allows for customization based on specific mine characteristics, making it applicable across diverse mining contexts. It provides a pathway for mining companies to engage in sustainable practices, reducing their carbon footprint while maintaining profitability. This aligns with global sustainability goals and contributes to the broader discourse on responsible resource extraction.

Future research should focus on validating this model across different mining environments and refining its parameters to enhance its predictive accuracy. By conducting case studies and exploring diverse carbon policy impacts, the model’s utility can be expanded, offering a valuable resource for the mining industry in its transition toward a sustainable future. Given the complexity of multi-parameter interactions, a more comprehensive sensitivity analysis that includes simultaneous changes in key parameters—such as gold prices, cut-off grades, and carbon tax rates—could provide valuable insights into how mining companies can optimize their strategies in response to varying market and policy conditions. However, such an analysis goes beyond the scope of this study due to its complexity and the challenges involved in modeling multiple interacting variables. Therefore, exploring the joint effects of these parameters will be a topic for future research.

## Conclusion

This research presents a System Dynamics (SD) model designed to support operational decisions for the low-carbon transition in underground gold mines. The model, grounded in statistical regression and dynamic simulation, enables both technical and economic analysis across various mining subsystems, including reserves, mining, ore dressing, smelting, finance, and carbon reduction. Applied to a gold mine in China, the model features causal loop diagrams and corresponding flow diagrams that illustrate the interrelationships among key system variables. The mathematical relationships derived from historical production and management data provide a clear representation of the system dynamics, with simulation results indicating that the accumulated profit of the gold mining enterprise over its service life will reach 1.51 × 10¹⁰ RMB.

The research demonstrates that the SD model can be effectively applied to mining enterprise decision-making in the context of carbon tax policies, successfully balancing economic profits with environmental benefits. A key innovation of this study is the integration of dynamic simulation with carbon emission reduction strategies, providing a powerful decision-support tool that allows mining enterprises to optimize resource utilization, extend the mine’s lifespan, and reduce carbon emissions while maximizing profitability. This model not only assists in economic optimization but also integrates sustainability goals within the decision-making framework, marking a significant step forward in the mining industry’s low-carbon transition.

The core contribution of this model lies in its ability to dynamically adjust to varying carbon tax policies, enabling mining companies to continuously optimize their carbon emission reduction strategies over time. This is particularly relevant in the face of evolving environmental regulations and market dynamics, offering mining enterprises a proactive approach to managing both economic and environmental outcomes. By allowing for real-time adjustments based on dynamic simulations, the model provides actionable insights that can lead to more sustainable and cost-effective mining practices.

It is important to note that the regression analysis, model construction, and parameter determination in this study were based on the actual production data of a specific mining area. Consequently, the immediate applicability of the model to other mines may be limited without recalibration using their unique operational data. However, the model’s flexible structure and adaptability to different mining contexts represent a significant advantage, as it can be customized and refined for diverse operational conditions, making it a valuable tool for the broader mining industry.

Future research should focus on overcoming the limitations of the current study by validating the model across various mining environments, analyzing its sensitivity to different carbon tax policies, and presenting case studies that showcase the model’s benefits in a wider range of mining contexts. These efforts will enhance the model’s relevance, extend its applicability, and attract broader attention, positioning it as a crucial resource for mining enterprises in their transition to a sustainable future.

## Data Availability

The datasets used and/or analysed during the current study available from the corresponding author on reasonable request.
